# Knowledge, attitude and practice of patients and their family members regarding age-related macular degeneration: a cross-sectional study

**DOI:** 10.3389/fpubh.2026.1811521

**Published:** 2026-04-22

**Authors:** He Zou, Danfeng Li, Xue Wang, Li Yu, Bo Yang, Lifu Luo, Jun Xiao

**Affiliations:** Department of Ophthalmology, The Second Hospital of Jilin University, Changchun, China

**Keywords:** age-related macular degeneration, attitude, cross-sectional study, knowledge, practice

## Abstract

**Introduction:**

Age-related macular degeneration (AMD) is a leading cause of irreversible vision loss among the older adults, and understanding the knowledge, attitudes, and practices (KAP) of patients and their family members is essential for improving disease management and patient education. This study aimed to assess the knowledge, attitudes, and practices (KAP) of patients with age-related macular degeneration (AMD) and their family members.

**Methods:**

A cross-sectional survey was conducted between April and July 2023 at the Department of Ophthalmology, the Second Hospital of Jilin University, using a self-administered questionnaire.

**Results:**

A total of 538 valid questionnaires were included in the analysis, comprising 325 patients (60.41%) and 213 family members (39.59%). Among all participants, 495 (92.01%) reported having undergone intraocular injection therapy, while 43 (7.99%) had not received such treatment. The mean KAP scores were 10.99 ± 1.30 for knowledge, 43.05 ± 3.48 for attitude, and 22.36 ± 3.96 for practice. Significant positive correlations were observed between knowledge and attitude, knowledge and practice, and attitude and practice. Structural equation modeling further demonstrated that knowledge had a significant direct effect on attitude, and attitude had a significant direct effect on practice, with attitude fully mediating the relationship between knowledge and practice.

**Discussion:**

Overall, patients and their family members exhibited adequate knowledge of AMD, whereas attitude and practice levels were moderate. These findings highlight the need for targeted educational interventions to enhance AMD-related knowledge, foster positive attitudes through supportive patient–family interactions, and facilitate the translation of knowledge and attitudes into appropriate health-related behaviors, with particular emphasis on timely medical consultation and adherence to treatment.

## Introduction

Age-related macular degeneration (AMD) is a prevalent condition, impacting approximately 170 million individuals worldwide (1, 2). This progressive macular disease is characterized by distinct fundus manifestations, including macular area drusen and atrophy, subfoveal disciform scarring, and exudative or neovascular macular degeneration, which are employed in its diagnosis (3, 4). AMD stands as a leading contributor to severe, irreversible vision loss among older populations, rendering it a significant global cause of blindness (5).

The Knowledge, Attitude, and Practices (KAP) model holds significant relevance in healthcare for assessing the knowledge, attitudes, and behaviors of target populations in their engagement with healthcare information (6, 7). KAP’s core premise, asserting that knowledge influences attitudes, which in turn shape individual behaviors, provides a robust framework for understanding and addressing health-related challenges (8). Given the potential for severe visual impairment caused by AMD, especially among the older adults in China, and recognizing the influential role of family members in patient care, an investigation into the KAP of Chinese patients and their families could uncover critical health education needs and intervention opportunities (9). Such insights have the potential to enhance AMD management and prevention, raise societal awareness, facilitate early detection and treatment, and ultimately reduce the risk of vision loss among affected patients.

Prior research has predominantly emphasized genetic factors, risk factors, pharmacoeconomics, and preventive measures related to AMD (10), while another study has predominantly focused on treatment options (10). In addition, a specific study just primarily assessed AMD awareness among residents of Beijing (11). Whereas, this study takes a pioneering approach by comprehensively investigating the KAP of Chinese patients and their family members concerning AMD, which may fill a significant void in the current research arena, offering a unique perspective on AMD in the context of China. Recently, a cross-sectional study conducted in Tianjin, China focused on the knowledge, attitudes, and practices of patients with age-related macular degeneration specifically toward anti-VEGF treatment under a one-stop intravitreal injection model (12). That study enrolled 493 AMD patients and used structural equation modeling to examine relationships among KAP variables, reporting suboptimal knowledge but positive attitudes and proactive practices toward anti-VEGF treatment. However, it did not include family members or examine broader KAP beyond anti-VEGF treatment in other clinical settings, and it was limited to a single service model in one geographic area. Therefore, the present study contributes additional evidence by including both patients and their family members and by investigating KAP related to AMD more broadly in a different regional context. Therefore, this study aimed to investigate the KAP of Chinese patients and family members towards AMD.

## Materials and methods

### Study design and participants

This cross-sectional study was conducted between April and July 2023 at the Department of Ophthalmology, Second Hospital of Jilin University. Participants were recruited from the AMD chronic disease management registry of our department, which comprises patients diagnosed with AMD and managed at our institution. Patient contact information was obtained from this registry for project communication and questionnaire distribution. The study enrolled patients diagnosed with AMD at the center, meeting the inclusion criteria of having undergone at least one anti-VEGF treatment. Questionnaires were online distributed, and patient contact information was obtained from the AMD chronic disease management system in the department for project communication and questionnaire distribution. This study was approved by the Medical Ethics Committee of the Second Hospital of Jilin University [approval No. 2023(094)]. Informed consent was obtained from each research participant before complete the questionnaire.

### Questionnaire

The questionnaire independently designed by the research team based on the 2023 edition of the “Chinese Age-Related Macular Degeneration Treatment Guidelines” and took inspiration from questionnaire designs used by other researchers (10, 11, 13) (Supplementary Questionnaire). It was not directly adopted or modified from any single existing validated instrument. Following the completion of the questionnaire design, a pilot study was conducted involving the collection of 41 questionnaires. The results of the reliability and validity test indicated a high level of internal consistency, with Cronbach’s *α* measuring at 0.8902. To further confirm the structural validity of the questionnaire, a confirmatory factor analysis (CFA) was conducted. The Kaiser-Meyer-Olkin (KMO) value was 0.874 (*p* < 0.001), indicating adequate sampling. The CFA model fit indices were as follows: chi-square/degrees of freedom (CMIN/DF) = 1.976 (reference: 1–3 excellent), root mean square error of approximation (RMSEA) = 0.043 (reference: < 0.08 good), incremental fit index (IFI) = 0.920 (reference: > 0.8 good), Tucker–Lewis index (TLI) = 0.912 (reference: > 0.8 good), and comparative fit index (CFI) = 0.919 (reference: > 0.8 good), confirming satisfactory model fit and supporting the structural validity of the questionnaire across the knowledge, attitude, and practice domains.

The final questionnaire in Chinese has four dimensions. The first dimension gathers demographic information through single-choice questions, including patient/self-identification, the type of hospital for eye treatment, history of intraocular injection surgery, eye disease screening in the past year, and the affected eye. Multiple-choice questions cover common chronic diseases, prior use of age-related macular degeneration medications, and treatment methods, reported in percentages. The second dimension assesses knowledge with two parts consisting of 14 questions, scored as 1 point for a correct answer and 0 points for an incorrect or unclear response, resulting in a score range of 0–14 points. The third dimension measures attitudes with 12 questions using a five-point Likert scale, including both positive and negative attitude questions, with a total score range of 12–60 points. Finally, the fourth dimension focuses on practices, featuring ten questions. Six are scored on a Likert scale, ranging from “always” to “never,” with a score range of 6–30 points. The remaining four questions are open-ended, gathering information on screening for age-related macular degeneration, check-up frequency, knowledge sources, and barriers to treatment. A score above 70% represents sufficient knowledge, a positive attitude, and active practice. A score between 50 and 70% indicates moderate knowledge, attitude, and practice. Scores below 50% signify insufficient knowledge, a negative attitude, and inactive practice (14).

### Statistical analysis

The minimum required sample size was calculated using the sample size calculation formula, where p was assumed as 0.5, *δ* = 0.05, and *α* = 0.05.

$n={\left(\frac{Z_{1-\alpha /2}}{\delta }\right)}^{2}\times p\times (1-p)$

The final plan entails collecting a minimum of 480 questionnaires, considering an anticipated effective response rate of 80%. The study population comprised AMD patients and their accompanying family members registered in the AMD chronic disease management system of our department. As this was a registry-based convenience sample rather than a probability sample from a defined finite population, the sample size formula above was applied to ensure adequate statistical power for the planned analyses.

Statistical analysis was conducted using SPSS 26.0 (IBM Corp., Armonk, NY, United States) and AMOS 27.0 (AMOS IBM, USA). Continuous variables were described using mean ± standard deviation (SD), and between-group comparisons were performed using t-tests or analysis of variance (ANOVA). Categorical variables were presented as *n* (%). The Spearman correlation was employed to assess the correlations between knowledge, attitude, and practice scores. Hypotheses were confirmed through the application of structural equation modeling (SEM). The hypotheses under examination included the influence of knowledge on attitude, knowledge on practice, and attitude on practice. Model fit was evaluated using root mean square error of approximation (RMSEA), incremental fit index (IFI), Tucker–Lewis index (TLI), and comparative fit index (CFI). Two-sided *p* < 0.05 were considered statistically significant in this study.

## Results

A total of 565 questionnaires were initially collected. After excluding 27 questionnaires that shared the same IP address, had discrepancies in the responses to demographic questions (e.g., selecting “g. None” while choosing other options) in question 3: “Does the patient have the following common chronic diseases?”, and had discrepancies in the responses to treatment methods (e.g., selecting “d. None” while choosing other options) in question 6: “Has the patient undergone the following treatment methods?”, a total of 538 valid questionnaires were retained, resulting in a questionnaire validity rate of 95.2%. The Kaiser-Meyer-Olkin (KMO) value of 0.874 indicated that the questionnaire’s validity was adequate.

### Baseline characteristics and KAP scores

Among them, there were 325 patients (60.41%) and 213 (39.59%) family members. The majority, 406 participants (75.46%), regularly sought treatment for eye diseases in tertiary hospitals, and 495 (92.01%) had undergone surgical treatment involving intraocular injections. Of the participants, 277 (51.49%) had undergone eye disease screening in the past year, and 144 (26.77%) experienced eye problems in both eyes.

The mean knowledge, attitude and practice scores were 10.99 ± 1.30 (possible range: 0–14), 43.05 ± 3.48 (possible range: 12–60), and 22.36 ± 3.96 (possible range: 6–30), respectively. The majority of participants demonstrated sufficient knowledge (87.36%), while the proportions with sufficient attitudes and practices were relatively lower, at 53.90 and 58.74%, respectively (Supplementary Table S1).

Demographics did not significantly affect knowledge scores. Attitude scores differed among participants with distinct eye conditions (*p* = 0.025), and those screened for eye disease in the past year displayed more proactive practices (*p* = 0.035) (Table 1). Hypertension affected 243 participants (45.17%), and 480 (89.22%) used intraocular injections of anti-angiogenic agents for age-related macular degeneration (Supplementary Table S2).

**Table 1:** Demographic information (single-choice questions).

Variables	*N* (%)	Knowledge, mean ± SD	*p*	Attitude, mean ± SD	*p*	Practice, mean ± SD	*p*
*N*	538						
Total Score		10.99 ± 1.30		43.05 ± 3.48		22.36 ± 3.96	
Are you the patient?			0.114		0.604		0.183
Yes	325 (60.41)	10.89 ± 1.37		43.09 ± 3.34		22.49 ± 3.83	
No, I’m a family member of the patient	213 (39.59)	11.13 ± 1.16		42.98 ± 3.67		22.16 ± 4.15	
Type of hospital for regular eye treatment			0.087		0.686		0.183
Tertiary hospital	406 (75.46)	11.05 ± 1.26		43.12 ± 3.41		22.53 ± 4.04	
Secondary hospital	74 (13.75)	10.95 ± 1.23		42.78 ± 3.38		21.91 ± 3.43	
Other hospitals (including community clinics, health centers, private clinics, etc.)	58 (10.78)	10.56 ± 1.54		42.91 ± 4.04		21.72 ± 3.98	
Patient’s history of intraocular injection surgery for eye treatment:			0.956		0.915		0.868
Yes	495 (92.01)	10.99 ± 1.30		43.06 ± 3.46		22.34 ± 3.92	
No	43 (7.99)	10.93 ± 1.26		42.95 ± 3.70		22.60 ± 4.41	
Screened for eye disease in the past 1 year:			0.618		0.570		0.035
Yes	277 (51.49)	10.96 ± 1.32		43.10 ± 3.29		22.68 ± 4.01	
No	261 (48.51)	11.01 ± 1.27		42.99 ± 3.67		22.02 ± 3.88	
Eyes affected by the patient’s eye problems			0.380		0.025		0.231
Left eye	201 (37.36)	11.04 ± 1.37		43.33 ± 3.60		22.47 ± 3.89	
Right eye	193 (35.87)	10.89 ± 1.29		42.51 ± 3.46		21.96 ± 3.92	
Both eyes	144 (26.77)	11.03 ± 1.20		43.38 ± 3.24		22.74 ± 4.08	

### Distribution of KAP

The distribution of knowledge dimensions revealed that the three knowledge items with the highest correctness rates were as follows: “Delayed treatment of age-related macular degeneration may lead to blindness.” (K11) with 98.51%, “Patients with age-related macular degeneration should consume more fruits, vegetables rich in vitamin C, and high-quality protein such as fish.” (K14) with 98.33%, and “Wet age-related macular degeneration can be treated with intraocular injections of anti-angiogenic agents.” (K4) with 96.65%. The three items with the lowest correctness rates were “It is common to develop age-related macular degeneration after the age of 40.” (K6) with 28.25%, “Age-related macular degeneration may have a genetic component.” (K7) with 31.60%, and “Surgery is needed to restore vision loss caused by age-related macular degeneration.” (K10) with 52.97% (Table 2).

**Table 2:** Knowledge of age-related macular degeneration patients.

Items	*N* (Correct rate %)
1. Age is a risk factor for developing age-related macular degeneration.	478 (88.85)
2. Vision loss and seeing dark spots in front of the eyes can be early signs of age-related macular degeneration.	499 (92.75)
3. Age-related macular degeneration is divided into dry and wet forms, and there is currently no effective treatment for dry age-related macular degeneration.	338 (62.83)
4. Wet age-related macular degeneration can be treated with intraocular injections of anti-angiogenic agents.	520 (96.65)
5. Smoking has no impact on age-related macular degeneration.	432 (80.3)
6. It is common to develop age-related macular degeneration after the age of 40.	152 (28.25)
7. Age-related macular degeneration may have a genetic component.	170 (31.6)
8. Blood tests can diagnose age-related macular degeneration.	490 (91.08)
9. Fundus angiography and OCT are both good methods for diagnosing eye health. OCT is more widely used for diagnosis and follow-up due to its non-invasive nature.	520 (96.65)
10. Surgery is needed to restore vision loss caused by age-related macular degeneration.	285 (52.97)
11. Delayed treatment of age-related macular degeneration may lead to blindness.	530 (98.51)
12. Patients with age-related macular degeneration are advised to wear a sun hat and protect their eyes from intense sunlight when going outdoors.	519 (96.47)
13. Patients with age-related macular degeneration do not need to control their weight, blood pressure, and blood lipids.	450 (83.64)
14. Patients with age-related macular degeneration should consume more fruits, vegetables rich in vitamin C, and high-quality protein such as fish.	529 (98.33)

The attitudes of AMD patients are marked by notable trends. A significant 93.87% perceive AMD as a severe eye disease (A4), with 94.24% believing it significantly disrupts daily life (A5). Moreover, an overwhelming 98.32% recognize the importance of prompt symptom response (A6). Patients also underscore the importance of lifestyle modifications, with 91.83% either strongly agreeing or agreeing to dietary adjustments and regular exercise (A7). High levels of trust in doctors’ AMD treatment plans are evident, with 94.61% expressing confidence (A8). Furthermore, patients emphasize preventive measures, as 96.93% endorse AMD screening for middle-aged and older individuals (A9), while 97.39% believe in regular post-treatment follow-up examinations (A10). Conversely, concerns exist, with 44.43% expressing fear of eye surgery (A11) and 67.48% voicing apprehensions about the financial burden of treatment (A12). These responses illuminate patients’ enthusiasm for learning, collaborating with healthcare professionals, and adhering to recommended lifestyle changes and treatments. Nonetheless, they also reveal nuanced facets, including apprehensions about eye surgery and financial considerations (Table 3).

**Table 3:** Attitude of age-related macular degeneration patients.

Items	Strongly agree	Agree	Neutral	Disagree	Strongly disagree
1. You are interested in learning about age-related macular degeneration.	240 (44.61)	215 (39.96)	81 (15.06)	2 (0.37)	0
2. You are willing to read literature, guidelines, and other professional materials to understand age-related macular degeneration.	213 (39.59)	239 (44.42)	84 (15.61)	2 (0.37)	0
3. You are willing to discuss age-related macular degeneration knowledge with healthcare professionals.	220 (40.89)	254 (47.21)	61 (11.34)	3 (0.56)	0
4. You consider age-related macular degeneration a severe eye disease.	281 (52.23)	224 (41.64)	27 (5.02)	5 (0.93)	1 (0.19)
5. You believe age-related macular degeneration greatly disrupts daily life.	294 (54.65)	213 (39.59)	27 (5.02)	4 (0.74)	0
6. You think it is necessary to seek medical attention promptly when experiencing symptoms suggestive of age-related macular degeneration (e.g., vision loss, distorted vision, dark spots).	341 (63.38)	188 (34.94)	9 (1.67)	0	0
7. You believe age-related macular degeneration patients should adjust their diet and engage in regular exercise.	229 (42.57)	265 (49.26)	39 (7.25)	5 (0.93)	0
8. You trust the treatment plans provided by doctors for age-related macular degeneration.	239(44.42)	270 (50.19)	29 (5.39)	0	0
9. You think it is necessary to conduct age-related macular degeneration screening for middle-aged and older individuals.	299 (55.58)	220 (40.89)	18 (3.35)	1 (0.19)	0
10. You believe age-related macular degeneration patients should undergo regular follow-up examinations after treatment.	285 (52.97)	239 (44.42)	14 (2.6)	0	0
11. You would feel extremely fearful if you needed eye surgery.	69 (12.83)	170 (31.6)	213 (39.59)	71 (13.2)	15 (2.79)
12. You believe that seeking hospital treatment would impose additional financial burden on your family.	144 (26.77)	219 (40.71)	112 (20.82)	50 (9.29)	13 (2.42)

When it comes to practice, a significant number of AMD patients (54.02%) actively seek information about their condition (P1), while 48.52% engage in constructive dialogues with healthcare professionals (P2). Furthermore, 80.66% diligently adhere to medical advice (P5), and 49.09% actively share their AMD knowledge (P8). In terms of hospital checkups (P2), 44.8% opt for monthly assessments. Diverse sources of knowledge (P3) are evident, with 68.96% relying on new media platforms and 63.01% seeking guidance from healthcare providers. Concerns related to AMD treatment (P4) are multifaceted, with the primary concern being recurrence and treatment duration (81.97%), followed by doubts about treatment effectiveness (67.47%). These findings collectively illuminate the proactive behaviors and complex considerations of AMD patients (Table 4 and Supplementary Table S3).

**Table 4:** Practices of age-related macular degeneration patients (single-choice questions).

Items	Always	Often	Sometimes	Occasionally	Never
1. You frequently seek information about age-related macular degeneration.	76 (14.13)	220 (40.89)	171 (31.78)	64 (11.9)	7 (1.3)
2. You regularly communicate with healthcare professionals to discuss the progression of your condition and inquire about protective measures.	71 (13.2)	190 (35.32)	203 (37.73)	65 (12.08)	9 (1.67)
5. You can follow medical advice to promptly undergo oral medication or intraocular injection therapy.	246 (45.72)	188 (34.94)	84 (15.61)	9 (1.67)	11 (2.04)
6. You pay close attention to adjusting your dietary habits.	122 (22.68)	224 (41.64)	155 (28.81)	26 (4.83)	11 (2.04)
7. You are vigilant about maintaining a healthy exercise routine.	143 (26.58)	229 (42.57)	126 (23.42)	33 (6.13)	7 (1.3)
8. You have shared knowledge about age-related macular degeneration with other family members, friends, and fellow patients.	93 (17.29)	171 (31.78)	197 (36.62)	65 (12.08)	12 (2.23)

### Correlation analysis and SEM

The correlation analysis conducted in this study revealed significant positive correlations among key variables. Specifically, a positive correlation was observed between knowledge and attitude (*r* = 0.1474, *p* = 0.0006), as well as between knowledge and practice (*r* = 0.1308, *p* = 0.0024). Additionally, attitude exhibited a positive correlation with practice (*r* = 0.4973, *p* < 0.001) (Supplementary Table S4).

**Figure 1:**
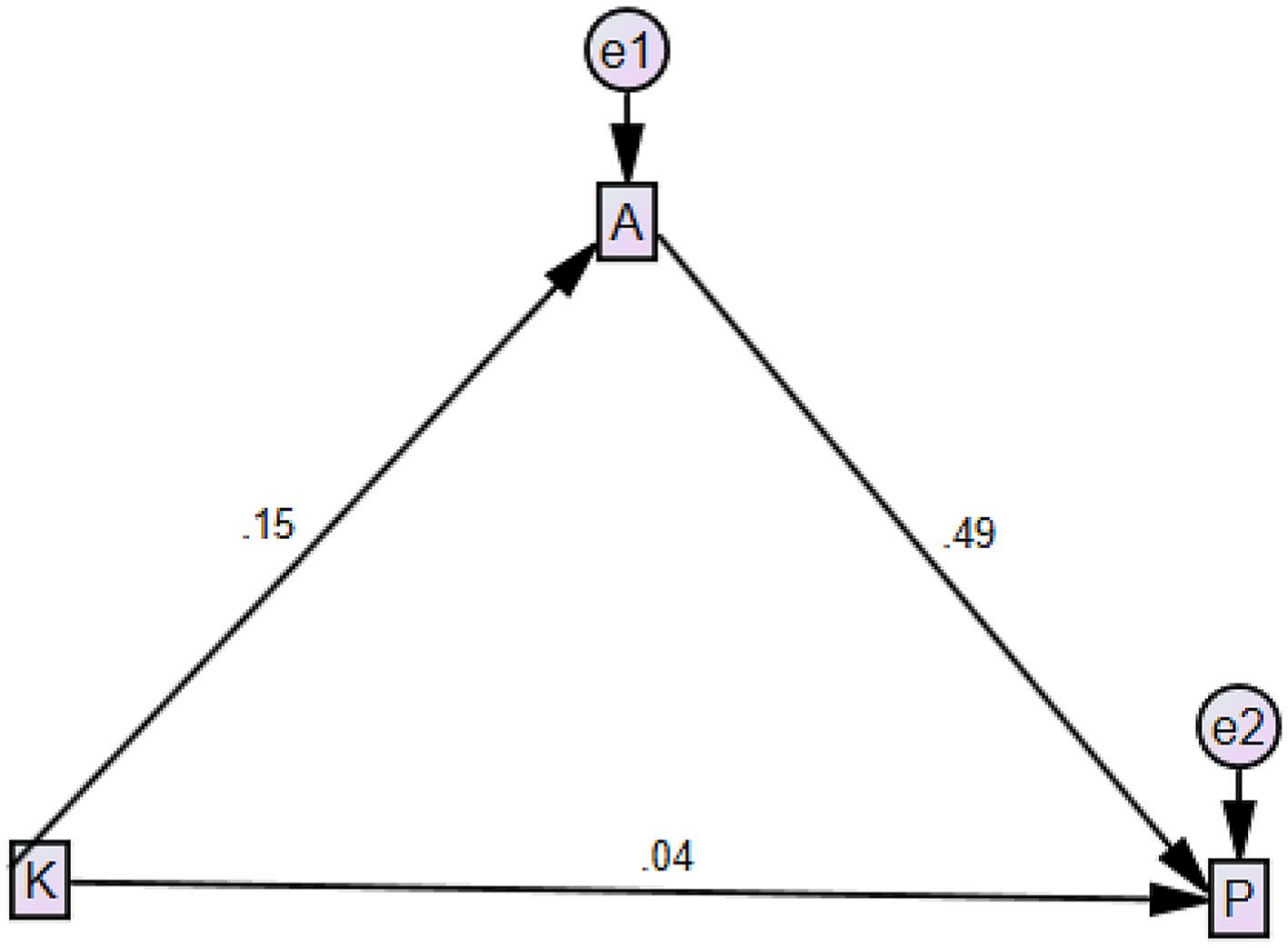
Structural equation model on KAP.

The SEM results further illustrated the relationships between variables: knowledge had a direct and significant effect on attitude (*β* = 0.15, *p* < 0.0001), while attitude had a direct and significant effect on practice (*β* = 0.49, *p* < 0.0001). The direct effect of knowledge on practice was not statistically significant (*β* = 0.04, *p* > 0.05) (Supplementary Table S5 and Figure 1).

## Discussion

Patients and their family members had adequate knowledge, positive attitude and proactive practice towards AMD. It is recommended that targeted educational initiatives aimed at improving AMD knowledge, the cultivation of positive attitudes through supportive interactions, and interventions to translate knowledge and attitudes into practical behaviors, with a specific emphasis on timely medical intervention and treatment adherence, be implemented.

The study results encompassed assessments of knowledge, attitude, and practice scores, each falling within their respective feasible ranges. Knowledge scores reflected a solid understanding of the subject matter (15), while attitude scores indicated a generally positive disposition toward relevant clinical practices (16). However, practice scores suggested a moderate level of adherence to recommended clinical practices. Recommended clinical practices in AMD management include regular follow-up examinations, strict adherence to anti-VEGF injection schedules, dietary modifications (e.g., increased intake of antioxidant-rich foods such as leafy vegetables and fish), UV protection (e.g., wearing sunglasses and hats outdoors), and control of systemic risk factors such as blood pressure and body weight, as outlined in the 2023 Chinese AMD Treatment Guidelines and supported by international evidence (14). The moderate practice scores observed in our study are consistent with findings from other KAP studies in chronic eye disease, which similarly report a gap between knowledge/attitude and actual health behaviors (15, 16). These findings collectively emphasize the need for focused interventions aimed at enhancing clinical practice, building upon the demonstrated knowledge and fostering a more robust alignment between attitude and practice, ultimately contributing to the improvement of overall healthcare quality (17, 18).

The attitude scores among participants with distinct eye conditions underscores the need for tailored interventions that consider the specific needs and perceptions of subgroups within the patient population. Furthermore, the observation that individuals who had undergone eye disease screenings in the past year exhibited more proactive practices underscores the importance of regular check-ups and the role of healthcare providers in reinforcing positive behaviors (19). Additionally, although laser therapy, photodynamic therapy, or transpupillary thermotherapy were once the means of treating AMD, in the last decade, anti-VEGF therapy has become the first-line choice for AMD treatment and has been included in many domestic and international clinical guidelines. To enhance clinical practice in the field of eye care, it is imperative to develop strategies that bridge the gap between knowledge and action, tailor interventions to address specific patient subgroups, promote regular screenings, and assess and address potential barriers to the utilization of essential therapies (20–22).

The study’s findings on patient attitudes towards AMD reveal important insights for improving clinical practice. Most patients view AMD as a severe eye disease, recognize its impact on daily life, and emphasize the importance of quick symptom response and trust in doctors’ treatment plans. They also show willingness to make lifestyle changes and support preventive measures like regular screenings and follow-up exams. However, concerns about eye surgery and financial burdens exist (23). These findings are broadly consistent with those of the Tianjin KAP study (15), which also reported positive attitudes toward AMD treatment among Chinese patients. However, in contrast to the Tianjin study, which focused specifically on anti-VEGF treatment attitudes, our study captures a broader attitudinal profile encompassing disease perception, lifestyle modification, and financial concerns. The high proportion of patients expressing fear of eye surgery (44.43%) and financial burden (67.48%) in our cohort is notably higher than reported in some Western studies (21), potentially reflecting differences in healthcare financing systems and cultural attitudes toward invasive procedures in the Chinese context. These results suggest the need for clear communication and patient education to reinforce positive attitudes, integrate holistic care approaches, and address specific patient concerns, ultimately enhancing clinical practice in AMD management (19). To further enhance patient care, it is crucial to implement comprehensive educational programs that provide detailed information about AMD, its progression, and available treatments. These programs should also address the emotional and psychological impact of the disease, offering support through counseling and support groups. Moreover, addressing financial concerns through transparent communication about treatment costs and available financial aid can help reduce the economic burden on patients. Educating patients about the long-term benefits of early intervention and consistent follow-up can motivate them to prioritize their eye health despite financial constraints.

The study’s findings regarding AMD patient practices reveal some key insights. Many patients actively seek information about their condition and engage in discussions with healthcare professionals. A significant majority diligently follow medical advice, and a substantial portion shares their AMD knowledge. Some patients opt for frequent hospital checkups. They obtain information from various sources, including new media platforms and healthcare providers (24). Concerns about recurrence, treatment duration, and treatment effectiveness are prominent. These practice patterns are broadly consistent with those reported in the Tianjin study (15), which similarly found that AMD patients in China demonstrate proactive information-seeking behaviors and generally adhere to medical advice. However, our study found that only 44.8% of participants opted for monthly hospital checkups, which is lower than the recommended follow-up frequency for patients receiving anti-VEGF therapy, suggesting that adherence to follow-up schedules remains a challenge. This diverges from the findings of Kamińska et al. (19), who reported higher rates of regular eye examination attendance in a Polish cohort, possibly reflecting differences in healthcare access and patient education between the two settings. These findings reflect the proactive approach of AMD patients in managing their condition and emphasize the importance of patient education and addressing specific treatment-related concerns in clinical practice (25).

No significant differences in KAP were found between patients and their family members, indicating the important role of family support in chronic disease management. An important and potentially innovative finding of this study is the inclusion of family members and the observation that their knowledge, attitudes, and practices did not significantly differ from those of patients. This suggests that family members may share similar perceptions of AMD and may actively participate in disease management. In the context of chronic eye diseases such as AMD, family members often play a key role in facilitating hospital visits, supporting long-term treatment adherence, assisting with daily care, and providing emotional support. Their comparable KAP levels indicate that educational interventions targeting only patients may be insufficient, and that family-centered education strategies could be more effective in reinforcing treatment compliance, promoting regular follow-up, and alleviating anxiety related to disease progression and financial burden. Therefore, integrating family members into AMD education and management programs may enhance the continuity and effectiveness of care. This finding aligns with evidence from chronic disease management literature, which consistently demonstrates that family involvement improves treatment adherence and patient outcomes (24, 25). To our knowledge, this is one of the few AMD KAP studies to include family members as a distinct study group, and the comparable KAP levels observed suggest that family-based educational interventions may be a promising and underutilised strategy in AMD management. Similarly, no significant differences were observed based on the type of hospital attended, suggesting uniform dissemination of information across healthcare settings. Our findings can be further contextualized by comparison with a recent cross-sectional study conducted in Tianjin, China (13), which investigated KAP of AMD patients specifically toward anti-VEGF treatment under a one-stop intravitreal injection model using structural equation modeling. That study reported relatively limited knowledge but generally positive attitudes and proactive practices toward anti-VEGF therapy. In contrast, the present study assessed KAP related to AMD more broadly, rather than focusing solely on anti-VEGF treatment, and included both patients and their family members. In addition, the Tianjin study was conducted within a specialized service model, whereas our participants were mainly recruited from routine clinical management settings in a different region of China. These differences in geographic location, service model, and study population may partly explain the variations in KAP patterns observed between the two studies and highlight the influence of regional healthcare organization and research scope on patients’ perceptions and behaviors. However, patients who had been screened for eye disease in the past year demonstrated significantly higher practice scores, emphasizing the importance of regular screenings in promoting proactive health behaviors. Interestingly, patients with both eyes affected by AMD had higher attitude scores, possibly due to the greater impact on their daily lives. The correlation and SEM analyses in this study unveil valuable insights into the interplay among key variables related to AMD management. The positive correlations observed between knowledge and attitude, knowledge and practice, as well as attitude and practice underscore the interdependency of these factors in AMD care (26, 27). Notably, the revised SEM results demonstrate that knowledge had a significant positive direct effect on attitude, and attitude had a significant positive direct effect on practice, while the direct effect of knowledge on practice was not statistically significant. These findings suggest that the influence of knowledge on practice is fully mediated by attitude, underscoring the pivotal role of attitude as a mediator in translating knowledge into health-related behaviors (28). Fostering positive patient attitudes therefore represents a critical step in facilitating the translation of knowledge into proactive AMD management practices (29, 30). These findings emphasize the need for comprehensive patient education strategies that not only impart knowledge but also cultivate favorable attitudes towards recommended AMD management, ultimately contributing to improved clinical practice and patient outcomes (31).

Limitations of this study include the reliance on self-administered questionnaires, which may introduce response bias. The patients were primarily from our tertiary hospital, diagnosed with AMD, and had received at least one anti-VEGF therapy, potentially limiting the depth and introducing further response bias. The cross-sectional design offers only a snapshot of knowledge, attitudes, and practices at a specific time, without establishing causality or capturing changes over time. Additionally, the sample population from a single university hospital’s ophthalmology department may not fully represent the diversity of AMD patients and their families in China, limiting the generalizability of the findings. Furthermore, the discrepancies observed in the understanding of medical concepts among patients highlight the need for improved patient education regarding AMD and its treatments.

## Conclusion

In conclusion, Chinese patients and their family members demonstrated adequate knowledge towards AMD, while attitude and practice levels were moderate. Targeted educational initiatives to improve AMD knowledge, the cultivation of positive attitudes through supportive interactions, and interventions aimed at translating knowledge and attitudes into practical behaviors, with a particular emphasis on timely medical intervention and treatment adherence, are recommended.

## Data Availability

The original contributions presented in the study are included in the article/Supplementary material, further inquiries can be directed to the corresponding author.
