# Supplementary material for: Knowledge, attitude and practice of patients and their family members regarding age-related macular degeneration: a cross-sectional study
Source: Front Public Health. 2026 Apr 22;14:1811521. doi: 10.3389/fpubh.2026.1811521 (PMC13144097; doi:10.3389/fpubh.2026.1811521)
Supplement: Supplementary file 1 [file Table_1.DOCX]

**Knowledge, Attitude and Practice of Patients and their Family Members Regarding Age-Related Macular Degeneration: A Cross-sectional Study**

| **Part.1 Demographic information** | | | | | | | | | | | | | | |  |
| --- | --- | --- | --- | --- | --- | --- | --- | --- | --- | --- | --- | --- | --- | --- | --- |
| 1. Are you the patient?： | | a. Yes  b. No, I’m a family member of the patient | | | | | | |  | | | | | |  |
| 2. Type of hospital for regular eye treatment： | | a. Tertiary hospital  b. Secondary hospital  c. Other hospitals (including community clinics, health centers, private clinics, etc.) | | | | | | | | | | | | |  |
| 3. Patient's history of intraocular injection surgery for eye treatment:： | | a. Yes  b. No | | | | | | | | | | | | |  |
| 4. Screened for eye disease in the past 1 year:： | | a. Yes  b. No | | | | | | | | | | | | |  |
| 5. Eyes affected by the patient's eye problems: | | a. Left Eye  b. Right Eye  c. Both Eyes | | | | | | | | | | | | |  |
| 6. Does the patient have the following common chronic diseases? (Multiple-Choice): | | a. Diabetes  b. Hypertension  c. Hyperlipidemia  d. Cardiovascular diseases  e. Fatty liver  f. Other  g. None | | | | | | | | | | | | |  |
| 7. Has the patient used the following medications for age-related macular degeneration? (Multiple-Choice): | | a. Intraocular injections - Anti-angiogenic drugs (e.g., Ranibizumab, Conbercept, Aflibercept)  b. Antioxidants (e.g., Oral Vitamin C, Vitamin E, Zinc, Lutein supplements)  c. Traditional Chinese Medicine (e.g., Compound Thrombolytic, He Xue Ming Mu Pian, etc.)  d. Other | | | | | | | | | | | | |  |
| 8. Has the patient undergone the following treatment methods? (Multiple-Choice): | | a. Laser therapy  b. Photodynamic therapy (PDT)  c. Transpupillary thermotherapy (TTT)  d. None | | | | | | | | | | | | |  |
| **Part.2 Knowledge Dimension** | | | | | | | | | | | | | |  |  |
| 1. Age is a risk factor for developing age-related macular degeneration. | | | | | | | a. Correct | | | | b. Wrong | | c. Unclear | |  |
| 2. Vision loss and seeing dark spots in front of the eyes can be early signs of age-related macular degeneration. | | | | | | | a. Correct | | | | b. Wrong | | c. Unclear | |  |
| 3. Age-related macular degeneration is divided into dry and wet forms, and there is currently no effective treatment for dry age-related macular degeneration. | | | | | | | a. Correct | | | | b. Wrong | | c. Unclear | |  |
| 4. Wet age-related macular degeneration can be treated with intraocular injections of anti-angiogenic agents. | | | | | | | a. Correct | | | | b. Wrong | | c. Unclear | |  |
| 5. Smoking has no impact on age-related macular degeneration. | | | | | | | a. Correct | | | | b. Wrong | | c. Unclear | |  |
| 6. It is common to develop age-related macular degeneration after the age of 40. | | | | | | | a. Correct | | | | b. Wrong | | c. Unclear | |  |
| 7. Age-related macular degeneration may have a genetic component. | | | | | | | a. Correct | | | | b. Wrong | | c. Unclear | |  |
| 8. Blood tests can diagnose age-related macular degeneration. | | | | | | | a. Correct | | | | b. Wrong | | c. Unclear | |  |
| 9. Fundus angiography and OCT are both good methods for diagnosing eye health. OCT is more widely used for diagnosis and follow-up due to its non-invasive nature. | | | | | | | a. Correct | | | | b. Wrong | | c. Unclear | |  |
| 10. Surgery is needed to restore vision loss caused by age-related macular degeneration. | | | | | | | a. Correct | | | | b. Wrong | | c. Unclear | |  |
| 11. Delayed treatment of age-related macular degeneration may lead to blindness. | | | | | | | a. Correct | | | | b. Wrong | | c. Unclear | |  |
| 12. Patients with age-related macular degeneration are advised to wear a sun hat and protect their eyes from intense sunlight when going outdoors. | | | | | | | a. Correct | | | | b. Wrong | | c. Unclear | |  |
| 13. Patients with age-related macular degeneration do not need to control their weight, blood pressure, and blood lipids. | | | | | | | a. Correct | | | | b. Wrong | | c. Unclear | |  |
| 14. Patients with age-related macular degeneration should consume more fruits, vegetables rich in vitamin C, and high-quality protein such as fish. | | | | | | | a. Correct | | | | b. Wrong | | c. Unclear | |  |
| **Part.3 Attitude dimension** | | | | | | | | | | | | | | | |
| 1. You are interested in learning about age-related macular degeneration. | a. Strongly agree | | | b. Agree | | c. Neutral | | | | d. Disagree | | e. Strongly disagree | | | |
| 2. You are willing to read literature, guidelines, and other professional materials to understand age-related macular degeneration. | a. Strongly agree | | | b. Agree | | c. Neutral | | | | d. Disagree | | e. Strongly disagree | | | |
| 3. You are willing to discuss age-related macular degeneration knowledge with healthcare professionals. | a. Strongly agree | | | b. Agree | | c. Neutral | | | | d. Disagree | | e. Strongly disagree | | | |
| 4. You consider age-related macular degeneration a severe eye disease. | a. Strongly agree | | | b. Agree | | c. Neutral | | | | d. Disagree | | e. Strongly disagree | | | |
| 5. You believe age-related macular degeneration greatly disrupts daily life. | a. Strongly agree | | | b. Agree | | c. Neutral | | | | d. Disagree | | e. Strongly disagree | | | |
| 6. You think it is necessary to seek medical attention promptly when experiencing symptoms suggestive of age-related macular degeneration (e.g., vision loss, distorted vision, dark spots). | a. Strongly agree | | | b. Agree | | c. Neutral | | | | d. Disagree | | e. Strongly disagree | | | |
| 7. You believe age-related macular degeneration patients should adjust their diet and engage in regular exercise. | a. Strongly agree | | | b. Agree | | c. Neutral | | | | d. Disagree | | e. Strongly disagree | | | |
| 8. You trust the treatment plans provided by doctors for age-related macular degeneration. | a. Strongly agree | | | b. Agree | | c. Neutral | | | | d. Disagree | | e. Strongly disagree | | | |
| 9. You think it is necessary to conduct age-related macular degeneration screening for middle-aged and elderly individuals. | a. Strongly agree | | | b. Agree | | c. Neutral | | | | d. Disagree | | e. Strongly disagree | | | |
| 10. You believe age-related macular degeneration patients should undergo regular follow-up examinations after treatment. | a. Strongly agree | | | b. Agree | | c. Neutral | | | | d. Disagree | | e. Strongly disagree | | | |
| 11. You would feel extremely fearful if you needed eye surgery. | a. Strongly agree | | | b. Agree | | c. Neutral | | | | d. Disagree | | e. Strongly disagree | | | |
| 12. You believe that seeking hospital treatment would impose additional financial burden on your family. | a. Strongly agree | | | b. Agree | | c. Neutral | | | | d. Disagree | | e. Strongly disagree | | | |
| **Part.4 Practice dimension** | | | | | | | | | | | | | | |  |
| 1. You frequently seek information about age-related macular degeneration. | | | a. Always | | b. Often | | | c. Sometimes | | | d. Occasional | e. Never | | |  |
| 2. You regularly communicate with healthcare professionals to discuss the progression of your condition and inquire about protective measures. | | | a. Always | | b. Often | | | c. Sometimes | | | d. Occasional | e. Never | | |  |
| 3.Have you ever undergone screening for age-related macular degeneration? | | | a. Yes | | b. No | | |  | | |  |  | | |  |
| 4. Do you regularly go to the hospital to check the effectiveness of your treatment? | | | a. Monthly checkups | | b. Checkups every 1-3 months | | | c. Checkups every 4-6 months | | | d. Yearly checkup | e. Never check | | |  |
| 5. You can follow medical advice to promptly undergo oral medication or intraocular injection therapy. | | | a. Always | | b. Often | | | c. Sometimes | | | d. Occasional | e. Never | | |  |
| 6. You pay close attention to adjusting your dietary habits. | | | a. Always | | b. Often | | | c. Sometimes | | | d. Occasional | e. Never | | |  |
| 7. You are vigilant about maintaining a healthy exercise routine. | | | a. Always | | b. Often | | | c. Sometimes | | | d. Occasional | e. Never | | |  |
| 8. You have shared knowledge about age-related macular degeneration with other family members, friends, and fellow patients. | | | a. Always | | b. Often | | | c. Sometimes | | | d. Occasional | e. Never | | |  |
| 9. How do you typically acquire knowledge about age-related macular degeneration? (Multiple-Choice) | | | a. Professional literature/guidelines | | b. Traditional media such as news, newspapers, and health programs | | | c. New media like WeChat public accounts (e.g., Small Molecule Care Home), TikTok, video apps, etc. | | | d. Explanations from professional healthcare providers or eye clinics | e. Other (e.g., information from neighbors, friends, or searching on Baidu) | | |  |
| 10. What do you consider as obstacles to receiving treatment for age-related macular degeneration? (Multiple-Choice) | | | a. Doubts about the treatment's effectiveness and uncertainty about whether it can cure the condition | | b. Limited financial resources and uncertainty about the total cost of treatment | | | c. Concerns about recurrence and uncertainty about the duration of treatment | | | d. Lack of a family member to accompany the patient, and family members being unaware that the patient's vision is impaired | e. Difficulty in accessing medical care at the hospital, such as difficulty in securing appointments and the hospital being too far away from home. | | |  |
