# Supplementary material for: Knowledge, attitude and practice of patients and their family members regarding age-related macular degeneration: a cross-sectional study
Source: Front Public Health. 2026 Apr 22;14:1811521. doi: 10.3389/fpubh.2026.1811521 (PMC13144097; doi:10.3389/fpubh.2026.1811521)
Supplement: Supplementary file 2 [file Table_2.DOCX]

**Supplementary Table 1 Distribution of knowledge, attitude, and practice (KAP) levels among participants (N = 538)**

| **Category** | **Knowledge (%)** | **Attitude (%)** | **Practice (%)** |
| --- | --- | --- | --- |
| Sufficient (>70%) | 470(87.36) | 290(53.90) | 316(58.74) |
| Moderate (50%-70%) | 68(12.64) | 248(46.10) | 212 (39.41) |
| Insufficient (<50%) | 0 | 0 | 10(1.86) |

**Values are presented as n (%).**

**Supplementary Table 2 Demographic and clinical characteristics of participants based on multiple-choice questions (N = 538)**

| **Question** | **N(%)** |
| --- | --- |
| **Does the patient have the following common chronic diseases? (Select all that apply)** |  |
| Diabetes | 90(16.73) |
| Hypertension | 243(45.17) |
| Hyperlipidemia | 79(14.68) |
| Cardiovascular diseases | 91(16.91) |
| Fatty liver | 61(11.34) |
| Other | 32(5.95) |
| None | 190(35.32) |
| **Has the patient used the following medications for age-related macular degeneration? (Select all that apply):** |  |
| Intraocular injections - Anti-angiogenic agents (e.g., Ranibizumab, Conbercept, Aflibercept) | 480(89.22) |
| Antioxidants (e.g., Oral Vitamin C, Vitamin E, Zinc, Lutein supplements) | 135(25.09) |
| Traditional Chinese Medicine (e.g., Compound Thrombolytic, He Xue Ming Mu Pian, etc.) | 97(18.03) |
| Other | 46(8.55) |
| **Undergone the following treatment methods? (Select all that apply):** |  |
| Laser therapy | 66(12.27) |
| Photodynamic therapy (PDT) | 6(1.12) |
| Transpupillary thermotherapy (TTT) | 3(0.56) |
| None | 465(86.43) |

Values are presented as n (%).

**Supplementary Table 3 Practice-related behaviors and perceptions among participants with age-related macular degeneration (N = 538)**

| **Items** | **N(%)** |
| --- | --- |
| **Have you ever undergone screening for age-related macular degeneration?** |  |
| Yes | 198(36.8) |
| No | 198(36.8) |
| **Do you regularly go to the hospital to check the effectiveness of your treatment?** |  |
| Monthly checkups | 241(44.8) |
| Checkups every 1-3 months | 167(31.04) |
| Checkups every 4-6 months | 42(7.81) |
| Yearly checkup | 67(12.45) |
| Never check | 21(3.9) |
| **How do you typically acquire knowledge about age-related macular degeneration?** |  |
| Professional literature/guidelines | 112(20.82) |
| Traditional media such as news, newspapers, and health programs | 156(29) |
| New media like WeChat public accounts (e.g., Small Molecule Care Home), TikTok, video apps, etc. | 371(68.96) |
| Explanations from professional healthcare providers or eye clinics | 339(63.01) |
| Other (e.g., information from neighbors, friends, or searching on Baidu) | 189(35.13) |
| **What do you consider as obstacles to receiving treatment for age-related macular degeneration?** |  |
| a. Doubts about the treatment's effectiveness and uncertainty about whether it can cure the condition | 363(67.47) |
| b. Limited financial resources and uncertainty about the total cost of treatment | 317(58.92) |
| c. Concerns about recurrence and uncertainty about the duration of treatment | 441(81.97) |
| d. Lack of a family member to accompany the patient, and family members being unaware that the patient's vision is impaired | 64(11.9) |
| e. Difficulty in accessing medical care at the hospital, such as difficulty in securing appointments and the hospital being too far away from home. | 104(19.33) |

Values are presented as n (%). Multiple responses were allowed for some items.

**Supplementary Table 4 Spearman correlation analysis among knowledge, attitude, and practice scores (N = 538)**

|  | **Knowledge** | **Attitude** | **Practice** |
| --- | --- | --- | --- |
| **Knowledge** | 1 |  |  |
| **Attitude** | 0.1474(P<0.001) | 1 |  |
| **Practice** | 0.1308(P= 0.002) | 0.4973(P<0.001) | 1 |

Values are presented as correlation coefficients (r) with corresponding P values. A two-sided P < 0.05 was considered statistically significant.

**Supplementary Table 5 SEM results (N = 538)**

| **Model paths** |  |  | Estimate | P |
| --- | --- | --- | --- | --- |
| Attitude | <--- | Knowledge | 0.147 | <0.0001 |
| Practice | <--- | Attitude | 0.492 | <0.0001 |
| Practice | <--- | Knowledge | 0.036 | 0.034 |

Values are presented as standardized path coefficients (β) and corresponding P values. SEM: structural equation modeling.
